# Effect of Demographic and Health Dynamics on Cognitive Status in Mexico between 2001 and 2015: Evidence from the Mexican Health and Aging Study

**DOI:** 10.3390/geriatrics6030063

**Published:** 2021-06-25

**Authors:** Silvia Mejia-Arango, Jaqueline Avila, Brian Downer, Marc A. Garcia, Alejandra Michaels-Obregon, Joseph L. Saenz, Rafael Samper-Ternent, Rebeca Wong

**Affiliations:** 1 Department of Population Studies, El Colegio de la Frontera Norte, Tijuana 22560, Baja California, Mexico; smejia@colef.mx; 2 Center for Alcohol and Addiction Studies, School of Public Health, Brown University, Providence, RI 02912, USA; jaqueline_avila@brown.edu; 3 Sealy Center on Aging, University of Texas Medical Branch, Galveston, TX 77555, USA; brdowner@utmb.edu (B.D.); almichae@utmb.edu (A.M.-O.); rasamper@utmb.edu (R.S.-T.); 4 Department of Sociology, Institute for Ethnic Studies, University of Nebraska, Lincoln, NE 68588-0324, USA; marcagarcia@unl.edu; 5 Leonard Davis School of Gerontology, University of Southern California, Los Angeles, CA 90089, USA; saenzj@usc.edu

**Keywords:** cognitive aging, epidemiology, healthcare disparities, minority health

## Abstract

Sources of health disparities such as educational attainment, cardiovascular risk factors, and access to health care affect cognitive impairment among older adults. To examine the extent to which these counteracting changes affect cognitive aging over time among Mexican older adults, we examine how sociodemographic factors, cardiovascular diseases, and their treatment relate to changes in cognitive function of Mexican adults aged 60 and older between 2001 and 2015. Self and proxy respondents were classified as dementia, cognitive impairment no dementia (CIND), and normal cognition. We use logistic regression models to examine the trends in dementia and CIND for men and women aged 60 years or older using pooled national samples of 6822 individuals in 2001 and 10,219 in 2015, and sociodemographic and health variables as covariates. We found higher likelihood of dementia and a lower risk of CIND in 2015 compared to 2001. These results remain after adjusting for sociodemographic factors, cardiovascular diseases, and their treatment. The improvements in educational attainment, treatment of diabetes and hypertension, and better access to health care in 2015 compared to 2001 may not have been enough to counteract the combined effects of aging, rural residence disadvantage, and higher risks of cardiovascular disease among older Mexican adults.

## 1. Introduction

Over the past 15 years, research on cognitive impairment and dementia in low- and middle-income countries has increased as the potential for a significant increase in dementia burden in faster-aging populations occurs [1]. Data from the Mexican Health and Aging Study (MHAS), a nationally representative panel study, has been widely used by researchers interested in cognitive aging. Several studies [2,3] have found that older age, fewer years of education, female sex, and rural residence are strong sociodemographic predictors of cognitive impairment. Highly prevalent chronic diseases among Mexican adults, such as hypertension, stroke, and diabetes, are associated with cognitive impairment [4,5].

In Mexico, it is well established that more recent cohorts of older adults aged 60 and older have higher educational achievement [6]. However, as a group, the more recent cohorts also have a higher prevalence of obesity, diabetes, hypertension, and physical disability [7,8]. This combination of change in key socioeconomic factors and health risks of the older adult population is quite evident by comparing cohorts who are only ten years apart, for example, those 60 and older in 2001 and 2012 [9,10,11]. Over the same decade, the country implemented reforms in social protection programs that benefit older adults, most notably the gradual implementation of Seguro Popular, a public health insurance program seeking to achieve universal health care, which started circa 2003 [12]. Previous work reports noticeable gains in the share of older adults with health insurance [13], the use of preventive care tests [4], and improved awareness and treatment of chronic conditions such as hypertension and diabetes [10]. Gender-specific associations have shown that the availability of health insurance is more likely to increase medical service utilization in women than in men [14].

This combination of relatively fast changes in sociodemographic and population health profiles of older adults likely contributed to changes in the burden of cognitive impairment and dementia. In high-income countries, despite increasing trends in cardiovascular health, previous studies report a decline in dementia incidence or prevalence among older adults, which they attribute largely to improvements in treatments of cardiovascular risk factors and rising levels of education [15,16,17]. In Mexico, it is unclear how the important sociodemographic and health-related changes previously mentioned, which occurred mostly in the last two decades [18], are associated with changes in cognitive aging among older adults. We seek to fill this gap by comparing data from MHAS collected at two different points in time: 2001 and 2015. 

The aim of this manuscript was to examine how sociodemographic factors, cardiovascular diseases, and their treatment relate to the cognitive status of Mexican adults aged 60 and older between 2001 and 2015. We hypothesized that higher educational achievement and more access to health care, including treatment of cardiovascular diseases, would imply a trend towards better cognitive function. On the other hand, survival to older ages and a higher prevalence of chronic diseases among survivors would indicate a trend towards worse cognitive function. These counter-acting influences mean that the overall time trend in cognitive function is ambiguous. Our approach sought to inform the extent to which the future burden of cognitive aging in Mexico can be potentially addressed with health interventions targeting better prevention, management, and treatment of highly prevalent chronic conditions. 

## 2. Materials and Methods

### 2.1. Participants

**Figure 1:**
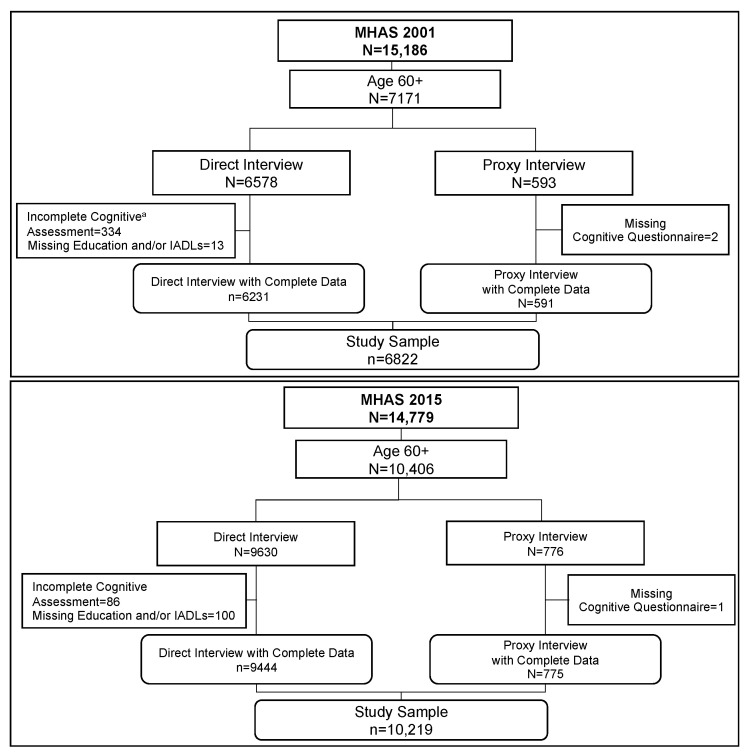
Flowchart of the Sample Selection for the 2001 and 2015 Cohorts. Note: Flowchart of sample selection in 2001 (*n* = 6822) and 2015 (*n* = 10,219). ^a^ More cognitive assessments were incomplete in 2001 compared to 2015 because more of the 2001 participants refused to participate in the cognitive assessment because they spoke only the Indigenous language. In 2015, these cases were no longer pursued.

The MHAS is a longitudinal cohort study of adults aged 50 and older in Mexico with national and urban-rural representation. The first wave was in 2001 with a sample of adults born in 1951 or earlier. Four follow-up waves were in 2003, 2012, 2015, and 2018 [19]. The study follows participants until their death. Additional refresher cohorts include those born in 1952–1961, added in 2012, and those born in 1962–1968, added in 2018. We used data from the 2001 and 2015 years for the current study [20]. The MHAS study seeks to complete interviews directly with the participants, but interviews by proxy are possible in cases of extreme disability, illness, or temporary absence. Figure 1 illustrates the sample selection process in both waves for the current analyses. We included all participants aged 60 and over who answered the survey through a direct or proxy interview. We selected individuals who completed at least two of five cognitive tasks included in the MHAS cognitive battery from those with a direct interview. Participants who refused the full cognitive assessment and those who completed only one task were excluded from our analysis (*n* = 334 in 2001 and *n* = 86 in 2015). The excluded participants tend to be male, older, and less educated. From proxy participants, we excluded those with incomplete information (*n* = 2 in 2001 and *n* = 2 in 2015). The final sample was 6822 participants in 2001 and 10,219 in 2015, which we treat as two cross-sections for our comparison. Individuals present in both cohorts were 2951, while 3871 were included only in 2001 and 8268 only in 2015.

### 2.2. Definition of Cognitive Categories

**Figure 2:**
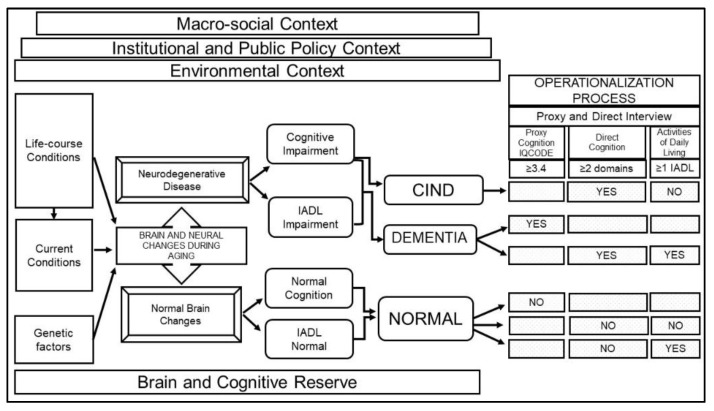
Conceptual framework for the definition of cognitive status in MHAS. Note: Framework showing how normal or pathological brain changes during aging are influenced by different factors (e.g., contextual, life-course, current conditions, genetic, brain reserve) and result in cognitive and functional symptoms (normal or impaired) which define cognitive status classification (CIND, dementia or normal cognition). Following this framework, data from MHAS is used to operationalize each category. Abbreviations: MHAS, Mexican Health and Aging Study; CIND, cognitive impairment no dementia; IADL, instrumental activities of daily living; IQCODE, Informant Questionnaire on Cognitive Decline in the Elderly.

A team of cognitive researchers collaborated with MHAS investigators to adopt a conceptual model for cognitive aging. We identified factors that influenced cognition and standardized the process to classify individuals into three cognitive categories: normal cognition, cognitive impairment no dementia (CIND), and dementia, using a comprehensive set of variables from the MHAS study. We summarize our framework in Figure 2 and highlight the established relationships between different factors that affect cognitive status. Figure 2 starts with three contextual levels in which cognitive aging occurs, namely: (1) The macro-social context, which includes the demographic and epidemiologic transitions; (2) The institutional and public policy context, which refers to the government and institutional support systems; and (3) The environmental context, which refers to group characteristics such as residence, population size, migration, and environmental exposures. These contextual aspects provide relevant information to understand potential differences between populations [21].

Underlying the process of cognitive aging are life-course socioeconomic and health conditions, current health conditions (cerebrovascular conditions, health behaviors, and health care), and genetic factors associated with normal or pathological (neurodegenerative disease) brain changes during aging [22]. Brain and cognitive reserve are key concepts to capture potential differences in the brain’s structural aspects and compensatory mechanisms that shape how the brain copes with age-related changes and pathology such as dementia [23]. These concepts include characteristics such as anatomical features of the brain structure, and the brain function indicators such as educational and occupational attainment. 

Following the criteria for preclinical and clinical phases of all-cause dementia recommended by the National Institute on Aging and the Alzheimer’s Association [24,25] and prior literature from population studies [26], we classified individuals into three groups based on their cognitive status and the ability to function independently in instrumental activities of daily living (IADLs). Individuals with a neurodegenerative disease may exhibit cognitive impairment and limitations in IADLs (dementia) or cognitive impairment, but no limitations in IADLs (CIND). On the other hand, individuals with normal brain changes will have normal cognition and no limitations in IADLs, or normal cognition and impairment in IADLs due to physical limitations.

The operationalization process linking these categories to the MHAS study variables was as follows: (1) Dementia: self-respondents with impairment in two or more cognitive domains and one or more IADL limitations, or proxy respondents with scores above the cut-point (≥3.4) on the Informant Questionnaire on Cognitive Decline in the Elderly (IQCODE) [27]; (2) CIND: self-respondents with impairment in two or more cognitive domains and no IADL functional limitations; (3) Normal cognition: self-respondents with normal cognitive function and no IADL limitations or impairment in IADLs due to physical limitations, and proxy respondents with IQCODE score below the cut-point (<3.4).

### 2.3. Cognitive Measures

The MHAS assessment in self-respondents uses a cognitive assessment adapted from the Cross-Cultural Cognitive Evaluation (CCCE) [28] in five cognitive domains: verbal learning (eight-word list presented during three trials), verbal memory (delayed recall of eight-word list), attention (visual scan of target stimuli), constructional praxis (copy of a figure), and visual memory (delayed recall of figure). Cognitive impairment in each domain was defined as a score 1.5 standard deviations (SD) below the mean based on reference norms by age and years of education [29]. The MHAS uses the adapted CCCE mainly because of its cross-cultural attributes, including ease of application among low-education groups. 

For respondents represented by a proxy, cognitive function was assessed through the brief version of the IQCODE [27], a 16-item questionnaire on cognitive decline in the elderly, rated on a 5-point scale from 1 “much improved” to 5 “much worse.” An informant who knew about the participant’s daily functioning, usually a spouse or adult child or caregiver, rated the participant’s cognitive status compared to how it was two years earlier. We used a cut-point of 3.4 and above to classify dementia, as the scale has a reported sensitivity of 85% and a specificity of 80% in population-based cohorts [30]. 

### 2.4. Covariates

Following previous research [3,8,18,26,31,32], we included the following covariates shown to have an association with cognitive impairment. Sociodemographic variables: sex (male/female); age used as a continuous or categorical variable (60 to 74 years and ≥75 years); years of formal education as a continuous or categorical variable (0 years, 1 to 6 years, and ≥7 years) following the formative periods in the Mexican education system; and area of residence as a categorical variable (rural for ≤2500 people and urban >2500 people). Cardiovascular risk factors: Self-reported stroke, diabetes, heart disease, and hypertension (all yes/no). Cardiovascular treatment: Among those who have each condition, self-reported use of medications for stroke, diabetes (either oral medications or insulin), heart disease, and hypertension (all yes/no). Body mass index (BMI); calculated with self-reported weight [kilograms] divided by height [meters and centimeters] squared [kilogram per square meter] categorized as underweight (<18.5), normal (18.5–24.9), overweight (25.0–29.9), obese (≥30) based on the World Health Organization (WHO) classification. Health insurance: Self-reported dichotomous variable (yes-no). 

### 2.5. Statistical Analysis

For descriptive analyses, we examined differences in covariates between total participants in the 2001 and 2015 cohorts and stratified by cognitive status using a *t*-test or χ^2^ test as appropriate. Because education is a critical covariate of cognitive aging, and there are important gender differences in educational achievement in Mexico, we present the multivariate results for the total analytical sample and results stratified by sex. For multivariable analyses, we pooled data from both years (2001 and 2015) and estimated two logistic regression models. The first model included the dichotomous dependent variable indicating if an individual had dementia versus no dementia (normal cognition and CIND); in the second model, CIND versus normal cognition was the dependent variable. In each model, we included a linear trend variable with the value 0 in 2001 and 1 in 2015. An odds ratio less or greater than 1 in the trend variable would indicate a decrease or an increase, respectively, in the likelihood of dementia and CIND in 2015 compared to 2001. We estimated an unadjusted model with the trend variable only and a fully adjusted model including all covariates: sociodemographic factors, BMI, each of four cardiovascular diseases, treatment for each disease, and health insurance. We tested for interactions between each independent variable and the trend variable to assess if the effect of each covariate was different in 2015 compared to 2001. We used IBM SPSS Statistics, Version 25.0 (IBM Corp., Armonk, NY, USA) for statistical analyses.

## 3. Results

### 3.1. Trends in Cognitive Status

Roughly 70–80% of the sample was classified as normal cognition (70.6% in 2001 and 79.1% in 2015). CIND individuals represented 23.3% (95% Confidence Interval [CI], 22.2–24.4) of the sample in 2001 and 13.5% (95% CI, 12.8–14.3) in 2015 (*p* < 0.001). Those classified with dementia were 6.1% (95% CI, 5.5–6.7) in 2001 and 7.3% (95% CI, 6.8–7.9) in the 2015 sample (*p =* 0.002). Table S1 in the Supplementary Material shows the distribution of covariates by cognition status for both years. 

### 3.2. Trends in Sociodemographic Characteristics

Table 1 shows descriptive characteristics of Mexican adults aged 60 and older in the years 2001 and 2015. Results were weighted to represent the national populations of older adults in the corresponding year. Compared with 2001, the cohort for 2015 contained a significantly larger proportion of individuals aged 75 years and older and this cohort had a higher average age. The share of women increased from 52.4% in 2001 to 54.8% in 2015 (*p =* 0.002). On average, older adults in 2015 had one more year of education compared to those in 2001 (*p* < 0.001). The proportion of individuals with no schooling decreased from 36.0% in 2001 to 24.0% in 2015 (*p* < 0.001), and the share of participants with seven or more years of education increased from 13.7% in 2001 to 22.9% in 2015 (*p* < 0.001). The absolute increase in educational achievement years was larger for men than for women and larger for those aged 60–74 compared to those aged 75 and older (data not shown). The proportion of individuals living in rural or urban areas did not differ significantly between 2001 and 2015.

### 3.3. Trends in Cardiovascular Risks and Treatment

The prevalence of some, but not all, cardiovascular conditions among Mexican adults aged 60 or older was higher in 2015 compared to 2001. Diabetes increased from 16.8% to 24.9% (*p* < 0.001). Similarly, hypertension increased from 41.3% in 2001 to 50.3% in 2015 (*p* < 0.001). Conversely, the prevalence of stroke decreased from 3.5% in 2001 to 2.6% in 2015 (*p* = 0.002), whereas heart disease prevalence did not change significantly. Regarding treatment among older adults, the proportion of participants with hypertension treatment increased from 76.3% in 2001 to 85.9% in 2015 (*p* < 0.001). Similarly, diabetes treatment increased from 87.6% to 93.2% (*p* < 0.001). Heart attack and stroke treatment were similar in the two periods.

Based on BMI classification, the share of individuals in the normal range decreased from 38.7% in 2001 to 34.8% (*p* = 0.004) in 2015. The underweight percentage also decreased slightly, from 3.5% to 2.2% (*p* = 0.03). The fraction of overweight remained the same between the two periods, while obesity increased from 16.3% in 2001 to 20.1% in 2015 (*p* < 0.001). Compared with 2001, the proportion of individuals with health insurance increased significantly, from 56.9% to 90.4% in 2015 (*p* < 0.001).

### 3.4. Regression Results

Table 2 shows the results of a fully adjusted logistic regression model, for the overall sample and stratified by sex, with dementia (versus normal cognition or CIND) as the outcome variable, and using pooled 2001 and 2015 data. The trend variable in the first row of the table represents the adjusted odds ratio (OR) of dementia in 2015 compared with 2001. The unadjusted OR of the trend variable (results not shown) showed a significantly higher likelihood of dementia in 2015 compared to 2001 for the overall sample (OR [95% confidence interval]: (1.23 [1.08–1.39], *p* = 0.001) and for men (1.37 [1.12–1.69], *p* = 0.002), but not significantly so for women (1.13 [0.97–1.32], *p* = 0.118)). After controlling for the effect of sociodemographic factors, cardiovascular conditions, treatment of cardiovascular conditions, BMI, and health insurance, both the overall sample and males continued to show higher odds of dementia in 2015. For females, the odds of dementia were also higher, now reaching statistical significance. 

For the overall sample, being female (1.50 [1.32–1.72], *p* < 0.001), being 75 years or older (4.53 [3.97–5.17], *p* < 0.001), and low educational achievement were associated with a significantly higher odds of dementia. Compared to having seven or more years of education, individuals with no schooling (1.93 [1.56–2.40], *p* < 0.001) and those with one to six years of education (1.36 [1.11–1.66], *p* = 0.003) had a higher likelihood of dementia. Higher odds of dementia also were associated with a history of hypertension, diabetes, and stroke. Being overweight (0.72 [0.63–0.83], *p* < 0.001) or obese (0.63 [0.52–0.77], *p* < 0.001) was associated with lower odds of dementia compared to having normal BMI.

The results showed noticeable differences when stratified by sex. Older age and lower education were associated with higher odds of dementia for both males and females. Living in rural areas was associated with increased odds of dementia in males (1.36 [1.09–1.71], *p* = 0.006) but not in females. Odds were significantly increased for those with hypertension (1.55 [1.17–2.05], *p* = 0.002) and diabetes (1.68 [1.02–2.77], *p* = 0.04) in females but not in males, while stroke was significant only in males (3.80 [2.21–6.4], *p <* 0.001). Similar to the overall sample results, being overweight or obese was associated with lower odds of dementia in males and females compared to those with normal BMI. 

Regarding self-reported treatment, the effect of diabetes and heart attack treatment was not significant, although both were associated with lower odds of dementia; for hypertension treatment, the association was significant only in females (0.71 [0.54–0.94], *p* = 0.02) but not in males. Receiving treatment for stroke was associated with a significantly *higher* odds of dementia, in both males (2.13 [1.11–4.09], *p* = 0.02) and females (4.04 [2.18–7.46], *p <* 0.001). Having health care insurance was associated with lower odds of dementia in females (0.66 [0.54–0.82], *p <* 0.001) but not in males. 

We tested for an interaction effect between each covariate and the trend variable (results not shown), controlling for the main effects of all the other covariates. Hypertension treatment was associated with significantly lower odds of dementia in 2015 (0.51 [0.29–0.90], *p =* 0.02) compared with 2001 in females, but not in males. No other interactions with the trend variable were significant.

Table 3 shows the results of a logistic regression model for the overall sample and stratified by sex with CIND (versus normal cognition) as the outcome variable, using pooled 2001 and 2015 data. The unadjusted OR of the trend variable (results not shown) showed a significant decline in the likelihood of CIND between 2001 and 2015 for the overall sample (OR [95% CI]: (0.52 [0.48–0.56], *p* < 0.001), for men (0.62 [0.55–0.69], *p* < 0.001), and for women (0.43 [0.38–0.49], *p* < 0.001)). After controlling for all covariates, these effects of the trend variable remained the same. 

For the overall sample, the fully adjusted model showed that being male (1.33 [1.22–1.44], *p* < 0.001) and living in rural areas (1.36 [1.23–1.49], *p <* 0.001) were associated with higher odds of CIND. Having no formal schooling (0.83 [0.72–0.94], *p =* 0.006), being overweight (0.88 [0.80–0.96], *p* = 0.008) or obese (0.70 [0.62–0.79], *p* < 0.001), and having access to health care (0.85 [0.77–0.95], *p =* 0.004) were all associated with lower odds of CIND. The results stratified by sex showed noticeable differences. For males, the odds of CIND were higher in those aged 75 years and older (1.26 [1.11–1.44], *p* < 0.001), while for females, the odds of CIND were lower in the same age group (0.78 [0.68–0.91], *p* < 0.001). Having no formal schooling was significantly associated with lower odds of CIND in males (0.76 [0.63–0.91], *p* = 0.004) but not in females. Having health care insurance was associated with lower odds of CIND in females (0.82 [0.70–0.95], *p* = 0.01) but not in males. Other results were similar for men and women; the odds of CIND were significantly higher for rural residence in males (1.36 [1.19–1.54], *p* < 0.001) and females (1.36 [1.18–1.56], *p* < 0.001). Compared to those with normal weight, the odds of CIND were lower for obesity in males (0.75 [0.62–0.90], *p* = 0.002) and females (0.67 [0.56–0.79], *p <* 0.001). Of note, there were no significant effects (*p* > 0.05) on the odds of CIND associated with any of the cardiovascular health conditions or the treatment of these conditions for the overall sample or for the sample stratified by sex. 

We tested for an interaction effect between each covariate and the trend variable, controlling for the main effects of all the other covariates. We found significant interactions with the sociodemographic variables (sex, age, education, rural residence), implying that the effect of these variables on CIND was higher in 2015 compared to their effect in 2001. No other interactions were significant.

## 4. Discussion

Our data confirmed three important changes that occurred during the 14-year period that may have affected the burden of cognitive aging. The composition of older Mexican adults changed towards their being older and more educated, although a gender gap in education favoring men was still evident. Other major changes were a much higher level of health insurance coverage and a higher prevalence of chronic health conditions, all consistent with previous research [18,32,33]. Because of these changes, it is possible that participants in 2015 were more aware of cardiovascular conditions than participants in 2001.

With this context of sociodemographic, health conditions, and health care changes, we used the cognitive status classification to examine whether the likelihood of cognitive impairment was different in 2015 compared to 2001 among adults aged 60 and older stratified by sex, and the effect of these covariates. Our multivariate models showed a robust result of higher odds of dementia and lower odds of CIND for both men and women in 2015 compared to 2001. Factors associated with a higher likelihood of dementia, such as older age, some cardiovascular risks, and rural residence, were operating in both years. Other factors associated with lower odds of dementia counteracted, such as higher educational achievement, being overweight and obese, and having health insurance. After all these factors were taken into account, a higher likelihood of dementia remained in 2015 for both men and women. Further, our results showed that the effect of these factors on the likelihood of dementia was overall similar for both years. 

Our results for dementia stratified by sex also convey noteworthy differences regarding the covariates. The detrimental effects of rural residence and stroke history were significant only for men, while the effects of hypertension and diabetes were evident only for women. The beneficial influence of health insurance was significant for women but not for men, a finding which was consistent with previous literature [14]. This finding is also consistent with our result that hypertension treatment was higher only for women, and the effect appeared to be higher in 2015 compared to 2001. This set of results points to future research regarding gender disparities in the disease risks associated with dementia in Mexican men and women. 

Surprisingly, stroke treatment showed a significant association with higher odds of dementia in both males and females. Further analyses showed that the time following stroke was shorter (five years or less) among those with cognitive impairment who were using medication compared to those with no treatment, suggesting a higher rate of post-stroke complications [34].

The covariates for the likelihood of CIND were quite different from those for dementia: only sociodemographic factors such as rural residence and obesity were associated with higher likelihood of CIND, and none of the cardiovascular risks or their treatment showed significant effects. The beneficial role of health insurance was evident only for women, and that of more education only for men. Overall, our CIND results seem rather unstable, consistent also with previous research [35]; CIND is an intermediate or transition stage between normal cognition and dementia. Previous studies using population-based data have reported that CIND is affected by myriad factors [36], and its measurement is a recognized challenge [37]. The MHAS made changes to the cognitive assessment over time, which could potentially influence the assessment of CIND. To minimize the impact of these changes, though, we classified cognitive status using the five original cognitive domains from 2001 that were also included in 2015. Future research ought to use longitudinal data to understand better CIND and its measurement. 

Our study confirmed an increase in obesity between 2001 and 2015. As in previous studies [17,38], we found that overweight and obesity were associated with lower odds of CIND and dementia. This result has been interpreted as evidence of a protective effect of higher late-life BMI for dementia as opposed to mid-life obesity [39]. Others have remarked on the importance of BMI changes during the life cycle to assess this association [40,41]. 

Rural residence and lack of health insurance are well-established sources of health disparities in populations worldwide, particularly in low- and middle-income countries [3]. Our results confirm that these are important risk factors for a higher likelihood of both dementia and CIND. Regarding health insurance, our results showed a beneficial influence of health insurance in 2015 compared to 2001, particularly among women and among those aged 75 and older [14]. Future research should further examine the mechanisms through which the expansion in health insurance over the period could have benefitted the cognitive function of women more than that of men, including the possibility of better treatment and awareness of diseases among women [10]. 

Our results with Mexican older adults differ from the results reported by others using a comparable period in the United States. Using a similar approach, results from the Health and Retirement Study reported a decrease in the risk of dementia in the United States between 2000 and 2012 [17]. The authors commented that increasing educational attainment and better control of cardiovascular risk factors were associated with a lower risk of dementia over this period. For Mexico, the higher educational attainment we observed for 2015 was still considerably lower than the gains observed for consecutive cohorts of older adults in the United States and other developed countries. As Mexico experienced increasing rates of chronic diseases, a corresponding better control of the conditions was largely absent. Thus, preventing and treating these diseases represents a critical opportunity to reduce the prevalence of dementia, in addition to the educational gains that will continue to prevail in future cohorts entering old age. 

We found that being female, older age, no schooling, lack of health insurance coverage, and cardiovascular risk factors were major contributors to increasing an individual’s odds of dementia in 2001 and 2015, mirroring what others have reported for other countries [42]. Overall, we interpret our results to indicate that the individual risk of dementia for a person with a given set of traits—age, sex, education, rural/urban residence, cardiovascular risks, health insurance—was higher in 2015 than in 2001. Other factors that were not included in our models may help explain this time trend and could be the subject of future research. 

Our study has several limitations. Our cognitive status classification was not based on clinical diagnoses. The classification of cognitive status for individuals who have data collected in population surveys depends on the definitions used and the information captured by the survey instruments. We classified cognitive impairment in self and proxy respondents using two cognitive screening instruments (CCCE and IQCODE), which correctly identify individuals with normal cognitive function and dementia [28,43], while cognitive assessment of those with CIND remains more challenging [37]. Our data also rely upon self-reported measures to determine if participants have any cardiovascular conditions. This approach has the limitation that undiagnosed diseases may be present; the under-reporting of conditions has been described in previous studies [44].

Despite these limitations, we contribute to the conceptual definition of cognitive status using a population-based survey, with our analyses of national samples of older adults in Mexico, using the same cognitive assessments, and including direct and proxy interviews. These efforts represent a significant improvement in our understanding of the dynamics of cognitive aging in Mexico. Future research using additional MHAS data will benefit from our work and continue to add to our knowledge of this crucial public health concern in rapidly aging societies. Policies and programs could focus on the prevention of cardiovascular diseases and their treatment as a direct strategy to reduce the burden of cognitive aging in Mexico, particularly among rural populations.

## 5. Conclusions

Countries like Mexico with a rapidly aging population may not experience quick increases in dementia prevalence. The trajectory followed will depend on the prevalence of comorbidities, access to health care, and the treatment and management of such conditions, among other factors. Over the 2001 to 2015 period, older age and higher prevalence of cardiovascular risks were evident, concurrent with gains in education and health insurance coverage among older Mexican adults. These risk and beneficial factors likely produced counteracting effects, leading to a slightly higher risk of dementia by 2015.

## Figures and Tables

**Table 1:** Characteristics of participants aged 60 and older of the 2001 and 2015 cohorts ^a^.

	2001	2015	
	*n* = 6822	*n* = 10219	*p* Value
Age, y			
60–74 years	5224 (74.5)	7073 (72.5)	<0.001
≥75 years	1598 (25.5)	3146 (27.5)	<0.001
Mean (SD)	69.3 (7.5)	71.2 (7.9)	<0.001
Sex			
Male	3169 (47.6)	4496 (45.2)	0.002
Female	3653 (52.4)	5723 (54.8)	
Education, y			
No schooling	2138 (36.0)	2142 (24.0)	<0.001
1–6 years	3646 (50.3)	5541 (52.9)	0.31
≥7 years	1038 (13.7)	2536 (22.9)	<0.001
Mean (SD)	3.6 (3.9)	4.9 (4.5)	<0.001
Residence			
Urban	4936 (57.8)	7267 (60.5)	0.08
Rural	1886 (42.2)	2952 (39.5)	
Cardiovascular conditions			
Hypertension	2876 (41.3)	5354 (50.3)	<0.001
Diabetes	1188 (16.8)	2686 (24.9)	<0.001
Heart attack	298 (3.3)	477 (3.8)	0.37
Stroke	254 (3.5)	293 (2.6)	0.002
CVD Treatment ^b^			
Hypertension	2190 (76.3)	4738 (85.9)	<0.001
Diabetes	1030 (87.6)	2499 (93.2)	<0.001
Heart attack	211 (66.8)	341 (70.7)	0.95
Stroke	141 (53.4)	153 (46.9)	0.44
BMI			
18.5–24.9 (Normal)	2491 (38.7)	3511 (34.8)	0.004
<18.5 (Underweight)	158 (3.5)	185 (2.2)	0.03
25.0–29.9 (Overweight)	2983 (41.5)	4331 (42.9)	0.08
≥30 (Obese)	1183 (16.3)	2182 (20.1)	<0.001
Health insurance (yes)	4287 (56.9)	9376 (90.4)	<0.001

Notes: Characteristics are presented as *n* (%) unless otherwise indicated. Values in parenthesis are weighted % derived using sampling weights of the MHAS. ^a^ The reported *p* value is for χ^2^ or *t* test for a significant difference in proportion or mean between years. ^b^ Cardiovascular treatment for those with each condition. Abbreviations: BMI, body mass index; CVD, cardiovascular conditions; SD, standard deviation.

**Table 2:** Odds ratios for dementia, overall and stratified by sex ^a^.

	Overall		Males		Females	
	OR (95% CI)	*p* Value	OR (95% CI)	*p* Value	OR (95% CI)	*p* Value
Trend (2015 vs. 2001)	1.27 (1.10–1.47)	0.001	1.33 (1.09–1.69)	0.020	1.24 (1.03–1.49)	0.020
Sex, Female	1.50 (1.32–1.72)	<0.001	NA		NA	
Age, y						
60–74 years	1 [Reference]		1 [Reference]		1 [Reference]	
≥75 years	4.53 (3.97–5.17)	<0.001	4.72 (3.78–5.90)	<0.001	4.34 (3.68–5.13)	<0.001
Education, y						
0 years	1.93 (1.56–2.40)	<0.001	2.13 (1.49–3.05)	<0.001	1.81 (1.38–2.37)	<0.001
1–6 years	1.36 (1.11–1.66)	0.003	1.40 (1.00–1.95)	0.05	1.32 (1.02–1.70)	0.03
≥7 years	1 [Reference]		1 [Reference]		1 [Reference]	
Residence						
Urban	1 [Reference]		1 [Reference]		1 [Reference]	
Rural	1.04 (0.90–1.20)	0.57	1.36 (1.09–1.71)	0.006	0.87 (0.72–1.05)	0.16
Cardiovascular disease						
Hypertension	1.27 (1.00–1.61)	0.05	0.74 (0.46–1.20)	0.23	1.55 (1.17–2.05)	0.002
Diabetes	1.60 (1.08–2.36)	0.02	1.56 (0.82–2.98)	0.17	1.68 (1.02–2.77)	0.04
Heart disease	1.46 (0.94–2.27)	0.09	1.46 (0.76–2.81)	0.25	1.46 (0.79–2.68)	0.22
Stroke	2.18 (1.52–3.13)	<0.001	3.80 (2.21–6.54)	<0.001	1.55 (0.95–2.51)	0.08
BMI						
Underweight (<18.5)	1.46 (1.06–2.01)	0.02	1.68 (1.01–2.79)	0.04	1.36 (0.90–2.07)	0.14
Normal (18.5–24.9)	1 [Reference]		1 [Reference]		1 [Reference]	
Overweight (25.0–29.9)	0.72 (0.63–0.83)	<0.001	0.70 (0.55–0.88)	0.002	0.73 (0.61–0.88)	0.001
Obese (≥30.0)	0.63 (0.52–0.77)	<0.001	0.55 (0.38–0.82)	0.003	0.66 (0.52–0.83)	0.001
CVD Treatment ^b^						
Hypertension	0.83 (0.65–1.06)	0.15	1.33 (0.81–2.19)	0.25	0.71 (0.54–0.94)	0.02
Diabetes	0.91 (0.60–1.36)	0.65	1.10 (0.56–2.14)	0.77	0.80 (0.48–1.34)	0.41
Heart disease	0.99 (0.59–1.68)	0.98	1.08 (0.51–2.32)	0.82	0.93 (0.45–1.92)	0.86
Stroke	3.24 (2.08–5.05)	<0.001	2.13 (1.11–4.09)	0.022	4.04 (2.18–7.46)	<0.001
Health care insurance ^c^	0.75 (0.63–0.89)	0.001	0.94 (0.71–1.26)	0.72	0.66 (0.54–0.82)	<0.001

Note. ^a^ Adjusted odds ratios were derived using logistic regression models with pooled data from 2001 (*n* = 6791) and 2015 (*n* = 10,215) for the overall sample; 2001 (*n* = 3155) and 2015 (*n* = 4494) for males; 2001 (*n* = 3636) and 2015 (*n* = 5271) for females. The dependent variable was dementia compared to normal cognition or CIND. ^b^ Cardiovascular treatment for those with each condition. ^c^ Health care insurance for those with insurance. Abbreviations: OR = odds ratio; CI = confidence interval; BMI = Body Mass Index; CVD, cardiovascular disease; NA, not applicable.

**Table 3:** Odds ratios for CIND, overall and stratified by sex ^a^.

	Overall		Males		Females	
	OR (95% CI)	*p* Value	OR (95% CI)	*p* Value	OR (95% CI)	*p* Value
Trend (2015 vs. 2001)	0.53 (0.49–0.58)	<0.001	0.61 (0.54–0.69)	<0.001	0.46 (0.41–0.53)	<0.001
Sex, Male	1.33 (1.22–1.44)	<0.001	NA		NA	
Age, y						
60–74 years	1 [Reference]		1 [Reference]		1 [Reference]	
≥75 years	1.01 (0.92–1.12)	0.70	1.26 (1.11–1.44)	<0.001	0.78 (0.68–0.91)	0.001
Education, y						
0 years	0.83 (0.72–0.94)	0.006	0.76 (0.63–0.91)	0.004	0.89 (0.74–1.07)	0.23
1–6 years	0.95 (0.85–1.06)	0.38	1.00 (0.86–1.16)	0.97	0.90 (0.77–1.06)	0.22
≥7 years	1 [Reference]		1 [Reference]		1 [Reference]	
Residence						
Urban	1 [Reference]		1 [Reference]		1 [Reference]	
Rural	1.36 (1.23–1.49)	<0.001	1.36 (1.19–1.54)	<0.001	1.36 (1.18–1.56)	<0.001
Cardiovascular disease						
Hypertension	0.98 (0.83–1.14)	0.80	0.95 (0.76–1.19)	0.68	0.98 (0.78–1.22)	0.86
Diabetes	1.10 (0.83–1.46)	0.50	1.20 (0.82–1.75)	0.33	0.98 (0.63–1.52)	0.93
Heart disease	0.69 (0.46–1.05)	0.09	0.78 (0.47–1.29)	0.34	0.53 (0.25–1.11)	0.09
Stroke	0.71 (0.48–1.06)	0.09	0.61 (0.32–1.14)	0.12	0.80 (0.48–1.33)	0.40
BMI						
Underweight (<18.5)	1.05 (0.79–1.40)	0.72	1.19 (0.79–1.78)	0.39	0.94 (0.62–1.42)	0.77
Normal (18.5–24.9)	1 [Reference]		1 [Reference]		1 [Reference]	
Overweight (25.0–29.9)	0.88 (0.80–0.96)	0.008	0.89 (0.78–1.01)	0.07	0.86 (0.75–0.98)	0.03
Obese (≥30.0)	0.70 (0.62–0.79)	<0.001	0.75 (0.62–0.90)	0.002	0.67 (0.56–0.79)	<0.001
CVD Treatment ^b^						
Hypertension	0.97 (0.83–1.14)	0.77	1.02 (0.80–1.29)	0.84	0.95 (0.76–1.19)	0.70
Diabetes	0.98 (0.73–1.32)	0.91	0.92 (0.62–1.37)	0.70	1.07 (0.68–1.70)	0.74
Heart disease	1.18 (0.73–1.92)	0.48	1.06 (0.58–1.92)	0.84	1.49 (0.64–3.45)	0.35
Stroke	1.56 (0.92–2.64)	0.09	1.93 (0.90–4.11)	0.08	1.30 (0.60–2.83)	0.50
Health care insurance ^c^	0.85 (0.77–0.95)	0.004	0.87 (0.75–1.01)	0.07	0.82 (0.70–0.95)	0.01

Notes: ^a^ Adjusted odds ratios were derived using logistic regression models with pooled data from 2001 (*n* = 6382) and 2015 (*n* = 9464) for the overall sample; 2001 (*n* = 3012) and 2015 (*n* = 4214) for males; 2001 (*n* = 3370) and 2015 (*n* = 5250) for females. The dependent variable was CIND compared to normal cognition. ^b^ Cardiovascular treatment for those with each condition. ^c^ Health care insurance for those with insurance. Abbreviations: CIND = cognitive impairment no dementia; OR = odds ratio; CI = confidence interval; BMI = Body Mass Index. CVD, cardiovascular disease; NA, not applicable.

## Data Availability

Publicly available datasets were analyzed in this study. This data can be found here: www.MHASweb.org (accessed on 4 December 2020).

## Supplementary Materials

The following are available online at https://www.mdpi.com/article/10.3390/geriatrics6030063/s1, Table S1: Descriptive statistics of MHAS participants aged 60 and over in the 2001 and 2015 cohorts by cognitive status.
