# Supplementary material for: Effect of Demographic and Health Dynamics on Cognitive Status in Mexico between 2001 and 2015: Evidence from the Mexican Health and Aging Study
Source: Geriatrics (Basel). 2021 Jun 25;6(3):63. doi: 10.3390/geriatrics6030063 (PMC8293108; doi:10.3390/geriatrics6030063)
Supplement: Supplementary file 1 [file geriatrics-06-00063-s001.zip › geriatrics-1265061-supplementary.pdf]

Supplementary Table S1.

Summary: Table S1 shows the distribution of sociodemographic variables, cardiovascular diseases, BMI levels, treatment of cardiovascular conditions and health insurance in those with normal cognition, CIND, and dementia between 2001 and 2015.

Table S1. Descriptive statistics of MHAS participants aged 60 and over in the 2001 and 2015 cohorts by cognitive status

	Normal Cognition			CIND			Dementia		
	2001	2015		2001	2015		2001	2015	
N	4819 (70.6)	8085 (79.1)	***	1589 (23.3)	1383 (13.5)	***	414 (6.1)	751 (7.3)	**
Sex									
Male	2224 (46.2)	3449(42.7)	***	799 (50.3)	767 (55.5)	**	146 (35.3)	280 (37.3)	
Female	2595 (53.8)	4636 (57.3)		790 (49.7)	616 (44.5)		268 (64.7)	471 (62.7)	
Age, y									
60–74 years	3768 (78.2)	5877 (72.7)	***	1269(79.9)	963 (69.6)	***	187 (45.2)	233 (31.0)	***
≥75 years	1051 (21.8)	2208 (27.3)		320 (20.1)	420 (30.4)		227 (54.8)	518 (69.0)	
Mean (SD)	68.9 (7.2)	70.2 (7.5)	***	68.6 (6.8)	71.0 (7.3)	***	76.3 (9.5)	79.5 (9.4)	***
Education, y									
No schooling	1535 (31.9)	1551 (19.2)	***	412 (25.9)	317 (22.9)	+	191 (46.1)	274 (36.5)	**
1-6 years	2573 (53.4)	4407 (54.5)		890 (56.0)	753 (54.4)		183 (44.2)	381 (50.7)	*
≥7 years	711 (14.8)	2127 (26.3)	***	287 (18.1)	313 (22.6)	**	40 (9.7)	96 (12.8)	
Mean (SD)	3.6 (4.0)	5.3 (4.6)	***	3.8 (3.9)	4.3 (4.2)	***	2.5 (3.4)	3.1 (3.7)	**
Residence									
Urban	3548 (73.6)	5892 (72.9)		1109 (69.8)	864 (62.5)	***	279 (67.4)	511 (68.0)	
Rural	1271 (26.4)	2193 (27.1)		480 (30.2)	519 (37.5)		135 (32.6)	240 (32.0)	
Cardiovascular diseases									
Hypertension	2043 (42.5)	4254 (52.6)	***	623 (39.3)	672 (48.6)	***	210 (51.2)	428 (57.0)	+
Diabetes	808 (16.8)	2121 (26.2)	***	274 (17.3)	349 (25.2)	***	106 (25.8)	216 (28.8)	
Heart disease	224 (4.7)	357 (4.4)		43 (2.7)	59 (4.3)	*	31 (7.6)	61 (8.1)	
Stroke	152 (3.2)	184 (2.3)	**	46 (2.9)	26 (1.9)		56 (13.6)	83 (11.1)	
BMI									
Normal	1693 (35.2)	2613 (32.4)	**	607 (38.2)	542 (39.2)		191 (46.1)	356 (47.4)	
Underweight	96 (2.0)	126 (1.6)		39 (2.5)	27 (2.0)		23 (5.6)	32 (4.3)	
Overweight	2135 (44.3)	3476 (43.0)		697 (43.9)	595 (43.0)		151 (36.5)	260 (34.6)	
Obese	890 (18.5)	1860 (23.0)	***	244 (15.4)	219 (15.8)		49 (11.8)	103 (13.7)	
CVD Treatment[a]									
Hypertension	1550 (76.1)	3783 (89.0)	***	474 (76.2)	582 (86.6)	***	166 (79.0)	373 (87.1)	**
Diabetes	697 (86.3)	1982 (93.4)	***	239 (87.2)	321 (92.0)	+	94 (88.7)	196 (90.7)	
Heart disease	155 (69.8)	256 (71.7)		35 (81.4)	40 (67.8)		21 (67.7)	45 (73.8)	
Stroke	75 (49.3)	81 (44.0)		27 (58.7)	15 (57.7)		39 (69.6)	57 (68.7)	
Health insurance[b]	3121 (64.8)	7458 (92.2)	***	954 (60.1)	1237(89.4)	***	212 (51.2)	681 (90.7)	***

Notes: CIND, cognitive impairment no dementia; BMI, body mass index; BMI Normal, 18.5-24.9; BMI Underweight, <18.5; BMI Overweight, 25-29.9; BMI Obese, ≥30; CVD, cardiovascular condition. Characteristics are presented as n (%) unless otherwise indicated. [a] Cardiovascular treatment for those with each condition. [b] Health care insurance for those with insurance.
***p < .001, **p < .01, *p < .05, + p < .10.
